# Types of cell death in diabetic cardiomyopathy: insights from animal models: Types of cell death in diabetic cardiomyopathy

**DOI:** 10.3724/abbs.2024213

**Published:** 2024-12-25

**Authors:** Hongjiao Xu, Zhuang Yu, Jun Zhu, Haoran Liu, Xiangyuan Chen, Jihong Jiang, Minmin Zhu, Jinbao Li

**Affiliations:** 1 Department of Anesthesiology Shanghai General Hospital of Nanjing Medical University Shanghai 200080 China; 2 Department of Anesthesiology Shanghai General Hospital Shanghai Jiao Tong University School of Medicine Shanghai 200080 China

**Keywords:** diabetic cardiomyopathy, DCM, apoptosis, necroptosis, ferroptosis, cuproptosis, experimental model

## Abstract

Approximately one-tenth of the global population is affected by diabetes mellitus, and its incidence continues to rise each year. In China, 1.4 million patients die of diabetes-related complications every year. Additionally, approximately 26% of patients with diabetes develop diabetic cardiomyopathy, with heart failure being one of the main causes of death in these patients. However, early detection of diabetic cardiomyopathy has proven to be difficult in a clinical setting; furthermore, there are limited guidelines and targeted means of prevention and treatment for this disease. In recent years, several studies have provided evidence for the occurrence of various forms of regulated cell death in diabetic myocardial cells, including apoptosis, necroptosis, ferroptosis, and cuproptosis, which are closely linked to the pathological progression of diabetic cardiomyopathy. Although most research on diabetic cardiomyopathy is currently in the animal trial phase, the inhibition of these regulatory cell death processes can limit or slow down the progression of diabetic cardiomyopathy. Therefore, this review discusses the appropriate animal experimental models currently available for diabetic cardiomyopathy and evaluates the roles of apoptosis, necroptosis, ferroptosis, and cuproptosis in diabetic cardiomyopathy. We hope to provide new methods and ideas for future research in diabetic cardiomyopathy.

## Introduction

The prevalence of diabetes mellitus (DM) has rapidly increased worldwide, and DM has emerged as one of the most common chronic metabolic diseases of the century and poses an ongoing threat to human health. According to the Diabetes Atlas of the International Diabetes Federation [1], the global prevalence of DM among individuals aged 20–79 years in 2021 was estimated to be 10.5% (536.6 million people); additionally, this figure is projected to rise to 12.2% (783.2 million people) by 2045. China, in particular, has the highest number of DM patients in the world, with 140.9 million patients with DM in 2021 and an expected 174.4 million patients by 2045. In 2021, the global diabetes-related health expenditure (DRHE) was estimated to be USD 966 billion; this figure has increased 316% over the last 15 years and is predicted to increase to USD 1.054 trillion by 2045. In China, the DRHE was USD 165.3 billion in 2021, which was second only to that in the United States; furthermore, this figure is expected to rise to USD 193.1 billion by 2045.

The occurrence of associated complications is a major component that contributes to the overall disease burden of DM and should not be overlooked. Approximately one-third of patients with DM experience heart-related diseases; furthermore, 1.4 million patients die of diabetes-related complications every year in China [2]. Overall, patients with DM have a greater prevalence of chronic conditions than non-DM patients do; these conditions can include hypertension, chronic coronary syndrome, and congestive heart failure (CHF) [3]. In 1972, Rubler *et al*. [4] proposed the concept of diabetic cardiomyopathy (DCM), which refers to the occurrence of DM-related myocardial complications in the absence of coronary artery disease, valvular disease, or relevant cardiovascular risk factors and sequelae. Two years later, Kannel *et al*. [5] conducted a study that evaluated 5209 DM patients for up to 18 years. Consequently, they established that DM is a standalone risk factor for CHF; additionally, the incidence of CHF among DM patients was higher than that in non-DM patients (5-fold increase in women, 2.4-fold increase in men), even after being adjusted for other risk factors such as age, coronary heart disease, and hypertension. This hypothesis was then verified by several subsequent studies [ 6– 8] . In 2020, Zghebi *et al*. [9] further demonstrated that DM was closely associated with CHF in a study involving more than 630,000 people (Odds Ratio 2.12 in women, 2.27 in men).

Various studies have shown that 19%–26% of patients with DM develop heart failure (HF) [ 7, 8, 10] , whereas the prevalence of DM in HF patients is approximately 25%–40% [6]. Overall, the DCM can be divided into two stages. The early stage may be asymptomatic and is characterized by concentric left ventricular (LV) hypertrophy, increased myocardial thickness and stiffness, elevated atrial filling pressure, and impaired diastolic function. The late stage is characterized primarily by increased myocardial fibrosis, which can lead to further impairment of diastolic function, thereby resulting in contractile dysfunction. Patients in the late stage may develop clear symptoms of HF, such as breathing difficulty at rest, shortness of breath upon exercising, lower limb edema, dizziness, fatigue, arrhythmia, and angina [ 11, 12] . Recent studies have suggested that DCM may not be a singular type of disease with continuous stages but may instead consist of two distinct phenotypes: restrictive/HF with preserved ejection fraction (HFpEF) and dilated/HF with reduced ejection fraction (HFrEF) [13]. Several effective medical treatments have been reported for dilated DCM, all of which are based on treatment methods established for dilated cardiomyopathy. In contrast, evidence for the effective treatment of restrictive DCM remains scarce [14]. Overall, we believe that DCM should be defined as myocardial dysfunction characterized by LV hypertrophy and diastolic dysfunction, which may not be accompanied by a reduced ejection fraction. Evidence of diastolic dysfunction and myocardial hypertrophy detected by echocardiography is critical for the early diagnosis of DCM. In addition, an elevated myocardial performance index is an indicator of early echocardiographic changes in DCM; hence, evaluating the myocardial performance index may also have some value in the diagnosis of DCM [15].

At present, there is a scarcity of compelling medical evidence regarding the treatment of DCM and an even greater lack of comprehensive reports outlining targeted clinical guidelines for this disease.

## Animal Models of DCM

Research into the pathophysiological mechanisms of DCM is still ongoing, and it is important to establish a stable and representative animal model. Because this disease is a myocardial complication resulted from diabetes, most current animal studies use DM models, with a longer modelling time until myocardial damage occurs. Common experimental animal models currently used for DM include those for type I diabetes mellitus (T1DM) and type II diabetes mellitus (T2DM); nonetheless, these two models differ in their pathogenic mechanisms. T1DM models are primarily established through the chemical induction of pancreatic toxicity, with the most commonly used drugs for establishing this model being STZ [16] and alloxan [17]. Both drugs exhibit selective toxicity towards pancreatic β cells, which can result in damage to or necrosis of these cells; consequently, insulin secretion is reduced in these animal models, effectively mimicking T1DM. As neither STZ nor alloxan can induce insulin resistance *in vivo*, these drugs are unable to fully simulate the pathophysiology of T2DM. In addition to STZ and alloxan, other compounds with diabetogenic effects include vacor, dithizone, gold thioglucose, and monosodium glutamate [18]. Specific genetically modified rodents, such as OVE26 transgenic mice [19] with calmodulin overexpression, nonobese diabetic (NOD) mice [20] with autoimmune DM, and BB-Wistar rats [21], have also been used in T1DM studies. Animal models of T2DM are constructed primarily through dietary adjustments, with the most adopted method being the high-fat diet (HFD) [22]. The implementation of a HFD can closely simulate the pathological process of obesity and insulin resistance [23]. However, these animals must be maintained on an HFD for a long period before reaching the optimal blood glucose level required for experimental evaluation; therefore, many studies employ an HFD in conjunction with low-dose STZ or alloxan for the joint construction of T2DM models [ 24, 25] . There are also representative transgenic animal models for T2DM, such as leptin-deficient ob/ob mice, leptin receptor-deficient db/db mice [ 26, 27] , and ZDF (fa/fa) rats [28]. Importantly, owing to the well-established protective effects of estrogen in both humans and rodents, females are almost never utilized in the development of animal models of DM [ 19, 29, 30] .

As previously discussed, DCM is a type of myocardial dysfunction characterized by LV hypertrophy and diastolic dysfunction; DCM may not be accompanied by decreased systolic function and a reduced ejection fraction, or these symptoms may appear in the later stages of DCM. Hence, it is necessary to replicate these types of cardiac changes in animal models of DCM. Imaging methods, such as nuclear magnetic resonance and echocardiography, have been used to establish that both diastolic and systolic dysfunction appear relatively early in animal models of DCM, with diastolic function occurring earlier than systolic dysfunction [ 31– 33] ; this progression is consistent with the pathophysiology of DCM observed in humans. In recent years, scientists have broadened their understanding of DCM animal models by moving beyond solely considering the reduction in the ejection fraction and instead focusing on the detection of early diastolic dysfunction. Thus, specific classic indicators commonly used for evaluating diastolic function, such as the mitral ratio of peak early to late diastolic filling velocity, mitral deceleration time, and isovolumic relaxation time, have been considered ultrasound indicators for the successful construction of DCM models [34]. Considering that the aforementioned indicators can be influenced by the subjectivity of ultrasound slice selection, more objective evaluation parameters, such as heart and LV mass, interventricular septal thickness, diastolic LV posterior wall thickness, and LV fractional shortening, should also be incorporated into the criteria needed to establish an effective DCM animal model [35].

## Cell Death in DCM

**Figure 1:**
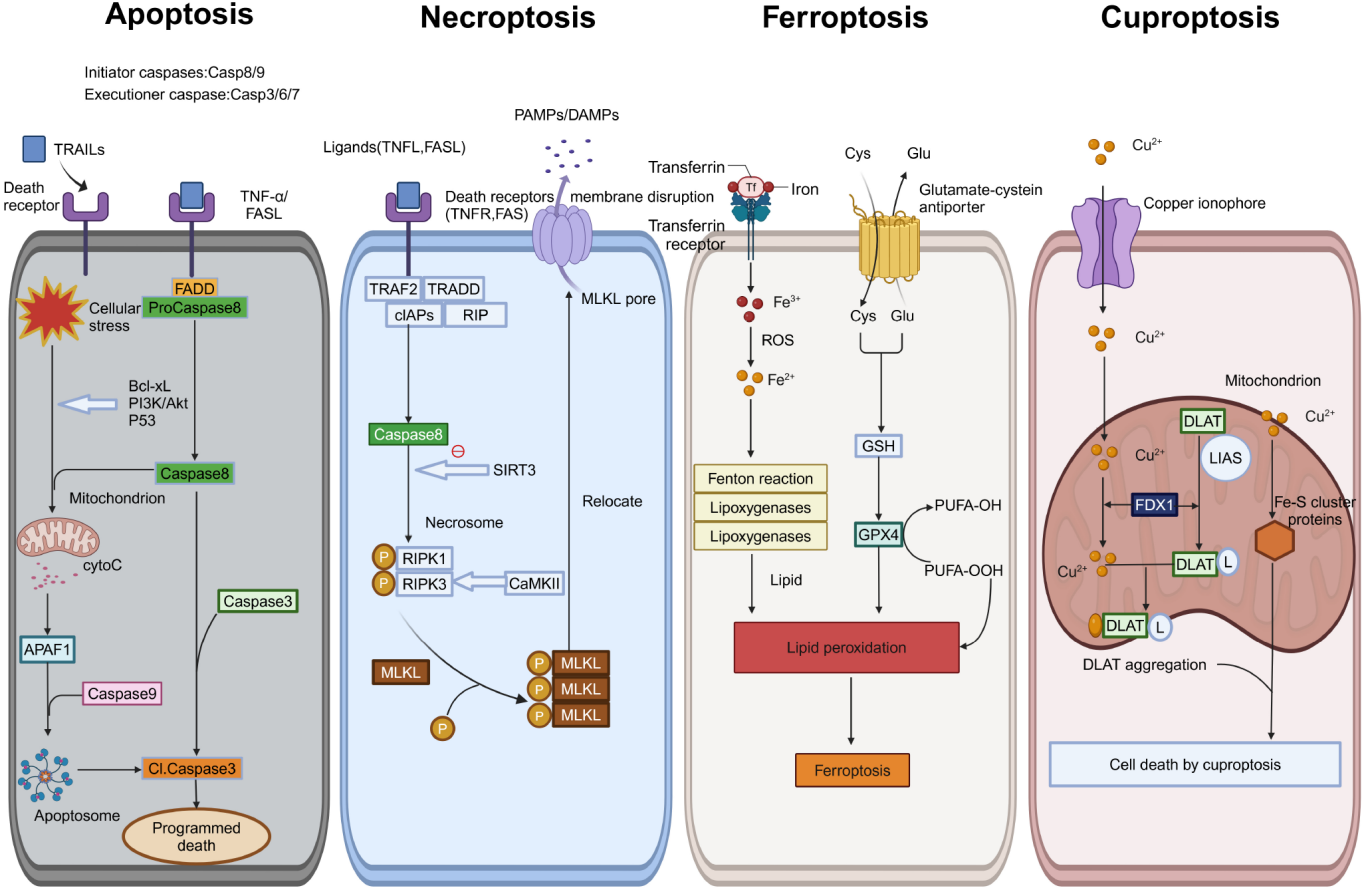
Figure 1 A microcosm of the different types of death in DCM, briefly delineates the activation mechanisms of the apoptosis, necroptosis, ferroptosis, and cuproptosis pathways

In recent years, a large body of evidence from animal experiments has established that DCM-induced cardiac dysfunction can be attributed to the dysregulated metabolism of cardiomyocytes. This dysregulation begins with insulin resistance but gradually develops into compensatory hyperinsulinemia, which eventually leads to hyperglycemia. This metabolic dysregulation can lead to various cardiometabolic abnormalities, including advanced glycation end-product deposition; excessive oxidative stress caused by the accumulation of reactive oxygen species (ROS); lipotoxicity; endothelial, microvascular, and mitochondrial dysfunction; extracellular matrix deposition; impaired intracellular calcium signaling; fibrosis; inflammation; overactivation of the renin-angiotensin-aldosterone system; cardiac autonomic neuropathy; and the unfolded protein response [ 4, 36– 41] . Collectively, these deleterious changes can lead to cardiomyocyte death, loss of myocardial mass, and interstitial fibrosis, which can result in impaired cardiac function and HF [ 42, 43] . Increased cardiomyocyte death is considered a major risk factor for the development of DCM. Kuethe *et al*. [44] evaluated endomyocardial biopsies of deceased patients and reported that cardiomyocyte apoptosis was 85 times greater in diabetic tissues than in nondiabetic tissues. Additionally, researchers have begun to turn their attention towards other DCM-related cell death pathways in addition to apoptosis [ 45, 46] . Correspondingly, this review aims to discuss the mechanisms underlying programmed cell death (apoptosis), programmed necrosis (necroptosis), ferroptosis, and cuproptosis of cardiomyocytes in patients with DCM. Additionally, we aim to elucidate the biological significance of these types of cell death under various pathological states ( Figure 1).

### Apoptosis

Since the concept of apoptosis was first proposed in 1972 [47], it has remained an active field of research over the last 50 years and has been established as a “basic biological phenomenon with wide-ranging implications in tissue kinetics” [48]. Cell apoptosis, also known as programmed cell death, is a process characterized by the cessation of cell growth and division, ultimately leading to cell death. A key morphological feature of apoptosis is that the contents of apoptotic cells do not leak into the surrounding environment. When cells detect damage or threats, initiator caspases (caspase 8 and caspase 9) are activated, which further regulate the activation of executioner caspases (caspase 3, caspase 6, and caspase 7). Ultimately, this initiates a series of reactions: DNA fragmentation caused by endonuclease activation, destruction of nucleoproteins and the cytoskeleton, protein cross-linking, expression of phagocytic ligands, and formation of apoptotic bodies [49]. Finally, the process of intrinsic apoptosis, otherwise known as the mitochondria-mediated apoptosis pathway, leads to the phagocyte-mediated engulfment of apoptotic bodies containing remnants of dead cells [50]. The extrinsic apoptosis pathway, otherwise known as the receptor-mediated apoptosis pathway, is induced by a combination of various death ligands and their corresponding receptors. Common ligands include members of the tumor necrosis factor-related apoptosis-inducing ligand family, such as tumor necrosis factor α (TNF-α) and fatty acid synthase ligands. After binding to their cognate receptors, these ligands can activate initiator caspases (caspase 8 and caspase 9), which in turn activate executioner caspases (caspase 3, caspase 6, and caspase 7), resulting in apoptosis [51]. The signal transduction process, which spans from the initiation of apoptotic signaling to the activation of apoptotic proteins, is regulated by various cytokines, including the B-cell lymphoma-2 (Bcl-2) family [ 52– 56] , the phosphatidylinositol 3 kinase/protein kinase B (PI3K/Akt) signaling pathway [ 57– 59] , and the P53 gene [ 60– 62] . Current methods for detecting apoptosis include flow cytometric analysis of Annexin V-FITC staining, DNA fragment analysis, and detection of TUNEL-positive cells. Furthermore, apoptotic bodies can be observed under a microscope during *in vitro* cell experiments.

Dysregulation of apoptosis has been implicated in the pathophysiology of various complications, such as tumors [62] and neurodegenerative diseases [63]. Many studies have demonstrated that long-term glycemia can induce cardiomyocyte apoptosis [64], which in turn plays a critical role in the pathogenesis of DCM and other DM-related heart diseases, such as myocardial infarction and HF [ 65, 66] . Cardiomyocyte apoptosis is a key pathogenic process of HF that can occur at any stage of this complication; specifically, the level of cardiomyocyte apoptosis can increase by up to 1000-fold in some models of HF [67]. Both the exogenous TNF-α-dependent apoptosis pathway and the endogenous mitochondria-dependent apoptosis pathway are involved in the cardiomyocyte apoptosis observed in DCM [ 68– 70] . Wu *et al*. [68] reported that the formation of mitochondria-associated endoplasmic reticulum membranes within a hyperglycemic environment promotes mitochondrial dysfunction and increases the degree of cardiomyocyte apoptosis in mice, which eventually results in the development of DCM. Interestingly, Chen *et al*. [71] reported that cardiomyocyte p-Akt expression does not increase after ischemia/reperfusion injury in diabetic rats but does increase in nondiabetic rats; alternatively, increasing p-Akt level leads to improvements in cardiac hemodynamic data, infarct size, and cardiomyocyte apoptosis in diabetic rats after ischemia/reperfusion injury. Furthermore, in this diabetic rat model, the expression levels of Bcl-2-associated X-protein (Bax) and cleaved caspase 3 decreased, whereas Bcl-2 expression and the Bcl-2/Bax ratio increased following ischemia/reperfusion injury.

### Necroptosis

Necroptosis is a regulated and typical form of programmed necrosis, or inflammatory cell death, characterized by several morphological features of necrosis, such as plasma membrane rupture, extensive swelling of the cytoplasm and organelles, and leakage of cellular components into the microenvironment [72]. Previously, researchers regarded necroptosis as necrosis rather than as a distinct type of cell death because of the overlapping features observed between the two types of death [73]. Only recently has necroptosis been proven to be a highly regulated process that acts as a protective mechanism for cells facing extreme microenvironments, such as infection. This discovery prompted further investigation into necroptosis as an independent form of cell death [ 72, 74] . Necroptosis is dependent on regulation by receptor-interacting protein kinases 1 and 3 (RIPK 1 and RIPK 3) and mixed lineage kinase domain-like protein (MLKL). Specifically, its chain of reactions is initiated by RIPK 1, which then binds to RIPK 3 through the RIP homotypic interaction motif domain to further recruit and phosphorylate MLKL [ 75– 77] . This ultimately results in the formation of a complex known as a necrosome, which mediates the occurrence of necroptosis.

Cao *et al*. [78] established that the cardiomyocytes of streptozocin (STZ)-induced type 1 diabetic mice actively expressed components of the RIPK 3/MLKL necrosis signalling pathway; ultimately, this confirmed the crucial role of necroptosis in the development of DCM. Recent studies have also demonstrated that sirtuin 3 is associated with the necroptosis of cardiomyocytes during DCM. Additionally, Song *et al*. [79] demonstrated that sirtuin 3 deficiency increased the expressions of the necroptosis-related proteins RIPK 1 and RIPK 3, which exacerbated DCM in diabetic mice. Conversely, Chen *et al*. [80] reported that sirtuin 3 activation could inhibit the onset of necroptosis via multiple pathways, thereby ameliorating DCM in a mouse model. As the third gasotransmitter in the cardiovascular system, hydrogen sulfide can exert protective effects on cardiomyocytes in DCM by reducing necroptosis [ 81, 82] . Chen *et al*. [83] detected high levels of apoptosis and necroptosis in the hearts of DCM mice. Overall, their findings indicated that cardiomyocyte necroptosis was triggered in the late stages of DM after apoptosis and that RIPK 3 was regulated by Ca ^2+^-calmodulin-dependent protein kinase II. Gao *et al*. [84] recently proposed the concept of a molecular feedback loop centered on cannabinoid receptor 2 (CB2R). Specifically, on the one hand, CB2R inhibits the S6 kinase-mediated phosphorylation of BTB domain protein and CNC homologue 2 (BACH2) at serine 520; this leads to the translocation of BACH2 to the nucleus, where it transcriptionally suppresses the expressions of necroptosis-related genes. On the other hand, hyperglycemia induces the internalization of CB2R; this enables the phosphorylation of CB2R at serine 352, which is mediated by MLKL and RIPK 3 (but not RIPK 1). Consequently, CB2R undergoes degradation via ubiquitin modification. Therefore, CB2R transcriptionally suppresses necroptosis via interactions with BACH2, whereas a decrease in MLKL activity results in negative feedback, which inhibits the phosphorylation and degradation of CB2R. Overall, these findings provide an integrative understanding of a novel molecular mechanism loop that regulates necroptosis, with CB2R as the central component.

### Ferroptosis

Ferroptosis is a regulated, iron-dependent type of cell death that is distinct from other forms of cell death. Specifically, it is characterized by lipid peroxidation and iron overload [85], with morphological features including reduced mitochondrial size, diminished mitochondrial cristae, and condensation or rupture of the mitochondrial outer membrane [86]. Ferroptosis is involved in the regulation of numerous mechanisms, such as infection, inflammation, immunity, tumorigenesis, and autophagy [ 87– 89] . Under the action of ROS and iron, polyunsaturated fatty acids in the cell membrane are converted into lipid hydroperoxides by the iron-containing enzyme lipoxygenase [ 90, 91] ; ultimately, these factors result in membrane damage associated with ferroptosis. Lipoxygenase is the main driver of ferroptosis; nonetheless, the function of this enzyme relies on the activation of acyl-CoA synthetase long-chain family member 4-dependent lipid biosynthesis [92].

In recent years, there has been a growing focus on developing novel treatment strategies for cardiomyopathy that specifically target ferroptosis [93]. Oxidative stress can disrupt the balance between the cellular antioxidant capacity and ROS generation, which is considered the core mechanism underlying the onset of DCM. Oxidative stress is also a key regulator of myocardial fibrosis, which in turn is an important feature of cardiac remodelling in DCM [94]. In addition, ROS accumulation promotes lipid peroxidation, which involves hydrogen atom abstraction from polyunsaturated fatty acids; this lipid peroxidation damages cardiomyocytes via the ferroptosis pathway [95] and accelerates the pathological progression of DCM. Ferroptosis-associated ROS include superoxide, hydrogen peroxide, and hydroxyl radicals; among these, hydroxyl radicals are considered the most active and toxic [96]. Nuclear factor erythroid 2-related factor 2 (NrF2) is a cytokine involved in multiple pathways of DM-associated cell damage; additionally, it has been recently implicated in the mechanism underlying cardiomyocyte ferroptosis in DCM [97]. Wang *et al*. [98] used sulforaphane to induce the activation of NrF2, which is mediated by AMP-activated protein kinase; this NrF2 activation suppressed ferroptosis signaling in cardiomyocytes and improved myocardial function in a DCM mouse model. Zhang *et al*. [99] demonstrated that NrF2 overexpression and nuclear translocation induced by curcumin can promote the expression of the oxygen scavenger heme oxygenase-1 (HO-1), ultimately inhibiting the development of cardiomyocyte ferroptosis in DCM. Additionally, Wu *et al*. [100] reported that 6-gingerol has cardioprotective effects on DCM mice via the NrF2/HO-1 signaling pathway. Furthermore, the sodium-glucose co-transporter inhibitor canagliflozin, which has been widely used in clinical practice as a glucose-lowering drug, has recently been proven to alleviate DCM via the ferroptosis pathway. However, current research into the role of ferroptosis in DCM has been conducted using only animal and cell models [ 101, 102] .

### Cuproptosis

Cuproptosis, a novel form of cell death, was recently discovered by Tsvetkov *et al*. [103]. This type of cell death is triggered by the targeted accumulation of copper within the mitochondria, which drives the direct binding of lipoylated tricarboxylic acid cycle enzymes to copper. Copper is an indispensable trace element in organisms; however, when the intracellular concentration of copper ions exceeds the threshold necessary for maintaining homeostasis, it can induce cytotoxicity [ 104, 105] . There is a large body of evidence demonstrating that the cell death associated with cuproptosis is mediated primarily by intracellular copper accumulation rather than by the direct accumulation of copper-binding small-molecule proteins. These proteins are only lethal when bound to copper via copper ionophores, and their cell-killing ability can be eradicated by modifications that eliminate their copper-binding capacity [ 103, 106] . Therefore, copper ionophores, which actively bind to and shuttle copper into the cell, have emerged as key research targets in the field of cuproptosis [107]. Cuproptosis, which is induced by copper ionophores, is a relatively unique mechanism compared with other known forms of cell death. Therefore, research on the mechanism of this newly discovered form of cell death is limited. Nonetheless, FDX1 is an upstream regulator of protein lipoylation, and its inhibition has been shown to block the tricarboxylic acid cycle [108]; this finding indicates that FDX1 may be a key factor in the cuproptosis pathway.

There are currently no available studies on the role of cuproptosis in DCM. Nevertheless, cardiomyocyte physiology has been established to be closely associated with copper metabolism. During the early 2000s, DM patients presented with abnormalities in copper metabolism. Compared with non-DM patients, patients with DM present elevated levels of copper ions in the plasma and urine but reduced copper levels in cardiomyocytes [ 109, 110] . Furthermore, cells that are more dependent on mitochondrial respiration are approximately 1000 times more sensitive to copper inducers than are cells that rely primarily on glycolysis. Thus, copper deficiency impairs mitochondrial function and leads to energy reduction, thereby resulting in myocardial hypertrophy [111]. Similarly, elevating copper levels in the blood can effectively ameliorate myocardial hypertrophy [112]. Zhang *et al*. [113] reported that structural and functional changes in the cardiomyocyte mitochondria of rats with diabetes-associated HF resulted in corresponding disruptions in copper homeostasis. Specifically, changes were observed in the levels of the mitochondria-resident copper enzymes cytochrome *c* oxidase and superoxide dismutase 1, alongside three major mitochondrial copper chaperones: cytochrome *c* oxidase copper chaperones 11 and 17 and copper chaperones for superoxide dismutase 1. Overall, their study demonstrated for the first time that dysregulated copper trafficking in cardiomyocytes may be a key pathogenic process in DCM, which aligns with previous theories in this field [114].

## Interference with Cell Death during Anaesthesia

Although surgical treatment is not a solution to DCM *per se*, patients with DCM face an increased risk of surgery. For example, metabolic surgeries such as Roux-en-Y gastric bypass and sleeve gastrectomy carry certain risks and can exacerbate the condition of diabetic cardiomyopathy patients. Moreover, surgery may further weaken immune function, thus increasing the surgical risk for patients with diabetic cardiomyopathy [115]. Current research indicates that perioperative anaesthetic drugs, including dexmedetomidine and propofol, can improve the increased perioperative risk associated with diabetic cardiomyopathy patients. Dexmedetomidine has been reported to reduce endoplasmic reticulum stress-induced cardiomyocyte apoptosis, thereby mitigating myocardial ischemia/reperfusion injury in DM rats [116]. Moreover, dexmedetomidine preconditioning activates the PI3K/Akt signaling pathway to reduce ischemia/reperfusion (I/R)-induced cell apoptosis, thereby providing cardiac protection against myocardial I/R injury in diabetic rats [117]. Furthermore, preconditioning cardiomyocytes with dexmedetomidine significantly reduces the cell apoptosis and oxidative stress induced by high glucose associated with hypoxia/reoxygenation (H/R) injury. Intraperitoneal injection of dexmedetomidine reduces the upregulation of apoptosis-related proteins and oxidative stress-related proteins in diabetic hearts, thereby mitigating I/R injury. Additionally, dexmedetomidine can mitigate diabetic myocardial I/R injury by increasing autophagy, reducing ROS production, and inhibiting inflammation through the HMGB1 pathway [118]. Propofol can mitigate mitochondrial damage and improve mitochondrial biogenesis by upregulating Cav-3 during hyperglycemia, thereby counteracting H/R-induced cell death in cardiomyocytes [119]. Postconditioning with propofol is achieved by upregulating FoxO1 and FoxO3a during hyperglycemia to mitigate H/R-induced apoptosis and autophagy in cardiomyocytes [120]. These studies suggest the rational use of anaesthetic drugs during surgery.

## Conclusions and Prospects

In current clinical practice, there are no specific prevention or treatment plans for DCM, which has been attributed to limited medical evidence. Correspondingly, there are no clear clinical guidelines, with the only recommended treatment for the onset of DCM-associated HF being based exclusively on HF guidelines. Despite the growing incidence of DM and the increasing number of DCM patients, the underlying mechanisms and clinical strategies for DCM prevention and treatment remain poorly understood; consequently, this knowledge gap has garnered increasing attention from researchers and clinicians. Owing to the limitations of human experiments, the appropriate selection of animal models is necessary for DCM research. Therefore, understanding and evaluating specific mechanisms of cardiomyocyte death through accurate experimental models may greatly benefit future studies into the underlying molecular mechanisms of DCM.

Once the blood glucose level is restored to normal, the continuous hyperglycemic stress state is characterized as hyperglycemic memory. Although the pathophysiological alterations induced by hyperglycemia-induced myocardial injury and those mediated by hyperglycemic memory exhibit similar pathophysiological changes, the underlying mechanisms might vary. Thus, focusing on the current mechanisms underlying the development of hyperglycemic memory could constitute a critical measure for exploring potential therapeutic targets for the treatment of diabetes-related complications.

In summary, the hyperglycemia-induced metabolic disorder DM can lead to multiple forms of cell death; additionally, various regulatory mechanisms of cell death have been implicated in the development of cardiomyocyte injuries in DCM. Therefore, the regulation of different cell death pathways may impact the progression of DCM; ultimately, this may serve as a foundation for novel ideas and objectives in future studies.
